# Hypodermic Medication

**Published:** 1875-06

**Authors:** J. B. Mattison

**Affiliations:** Chester, New Jersey


### HYPODERMIC MEDICATION.

BY J. B. MATTISON, M. D., OF CHESTER, NEW JERSEY.

I have used a hypodermic syringe for at least ten years, and with the happiest results; and further, without any accidents, except two casses of puffiness and inflammation, resolved without suppuration. I use it, invariably, in every case of acute pain, such as colic, or severe accident, or the invasion of pleuritis, or extraordinary after-pains, strangulated hernia, gravel, biliary calculi, etc. My reason is, that the absorbing functions of the alimentary canal, in common with others, are hindered, often suspended, by pain. That this is so, look at the parched mouth of a parturient woman. In these cases I use hypodermic to make a decided impression, and finish with the ordinary modes by the mouth, or rectum, or skin, as the case may demand. The syringe is useful also where the stomach is full of food, as just after a meal. I have used it twice also in cases of opium poisoning to antagonize the narcotine with atropine. In one the result was very happy; in the other it was unavailing. This last was a case of puerperal peritonitis, complicated with uræmic anasarca.

I have used both glass and hard rubber cylinders. The glass has only one advantage over the other; it enables one to see whether his syringe contains air. But this can also be ascertained, as to the hard rubber ones, which have these two advantages over the glass—the bore is truer, the joints are tighter and there is less leakage of air.

I shall take the liberty to give in detail the management of the syringe. First, it must be tight and smooth in its workings.—Before attaching the nozzle, the mouth of it must be closed with the finger, and the piston drawn out, and then released; if it flies back, the vacum is complete. Then draw out the piston and try the reverse experiment. Ascertain whether the piston can be pushed in permanently with the nozzle closed. If it is not in order, take it out and spread the packing with the fingers, taking care not to indent the edge of it with the nail, or it will surely leak. When in order, put on the nozzle tightly, and fill very slowly.—

Hold the instrument perpendicularly, nozzle up, tap it with the finger nail, and push in the piston slowly until it is certain that only liquid exudes. If any air has found its way within, replace it with the liquid. If the syringe is a new one, test its graduation by emptying it into a correct minim graduate. It will probably be erroneous, but this is of minor consequence, because a simple calculation gives, once for all, the number of minims corresponding to its divisions. Be sure that it is clean. I have heard of using it, in an emergency, as an exploring trocar. Such use would contaminate the packing beyond the possibility of *certain* cleansing, and expose the patient to the risk of septicæmia.

In introducing the nozzle, avoid the usual situation of venous of nerve trunks. Put the nozzle well into the areolar tissue, and then withdraw it a little, so that its end may be free, and that there may be a passage for the fluid along its side into the tissues. The finger ought to press on the nozzle at its entrance, to prevent exudation of the fluid.

I have been thus minute, because I have more than once known a practitioner disappointed, in consequence of throwing in more air than liquid, by being ignorant of the capacity of his syringe.

I keep always on hand two solutions, viz.: Magendie's solution of morphia sulph., and atrop. sulph. gr. ij., to the fluid ounce.—The acetates are a little more likely to become mouldy. By the way, I dispense morphia always as Magendie's solution, carrying with me a minim graduate, and thus add the requisite quantity to a definite number of actual teaspoonfuls of rain water. In making the solution, I put the one-eighth of an avoirdupois ounce into twenty-seven fluid drachms of *distilled* water.

Powers and Weightman's packages are very reliable, as containing fifty-four grains. This solution is apt to become a little mouldy, for it is impossible to keep distilled water free from germs, even if they did not go over in the distilling process. But if the morphia is present as a sulphate, it will not be impaired.—*Thomas Ryerson, M. D., Trans. N. J. State Medical Society*, 1874.

### *RAW COTTON DRESSING FOR WOUNDS.*

Prof. Wm. H. Van Buren, in a recent lecture, called attention to the above dressing, which, introduced in a Parisian hospital, during the Franco-German war, has since attracted a large amount of interest, especially abroad. It has long been used as an application for burns, but its employment in wounds, little and large, bids fair to prove immensely beneficial, if the enthusiastic enconiums lavished upon it by its advocates be worthily bestowed.

Pasteur and Tyndall have shown that air forced through the meshes of raw cotton, is deprived of its poisonous particles, which are assumed floating therein, and which, getting into the system through open wounds, favor the development of pyæmia, and that, therefore, this dressing becomes the most effective protection against the scourge of the hospital ward.

During the winter of 1870–1, when the hospitals in Paris were filled to overflowing, pyæmia appeared, and proved so disastrous that amputation was almost necessarily fatal. At this juncture, it occurred to Alphonse Gueren, surgeon of the St. Louis Hospital, that the assertion of Pasteur might prove advantageous, and he, accordingly, made use of the cotton dressing, applying it in liberal quantity, and bandaging firmly. To his delighted surprise, the premonitory chill ushering in the fatal complication failed to occur, and patients progressed to a speedy recovery. Encouraged, he repeated it with like success, and so remarkable were the results, wounds thus dressed, eventuating favorably, while those treated otherwise proved fatal, that other surgeons flocked to the St. Louis, and being firmly convinced, adopted it as a surgical dressing, and its employment soon became general.

Gueren claims, in communications to the French Academy, March 23d, and May 18, 1874, that it excludes effectually, poisonous germs from recent wounds, but *does not entirely exclude air*, and in proof of this statement, says he has found pus in contact with a wound undisturbed for thirty days, perfectly fresh and odorless, and while presenting evidence that oxygen had reached it, showing no signs whatever of putrefactive decomposition.

He applies the cotton very carefully, and does not remove it until a barrier of granulations has sprung up, or the wound has entirely healed. Too early a withdrawal exposes the patient to risk of pyæmic poisoning, as Guerin found by unpleasant experience, having in two cases that were doing perfectly well, removed the dressing after a week's application, and in three days this complication developed itself, and his patients succumbed.

Previous to applying, he washes the wound thoroughly with some germ killing-fluid, using indifferently, weak camphorated spirits or a solution of carbolic acid.

Under favorable circumstances the dressing is allowed to remain a fortnight, at the expiration of which the granulating surfaces are ready for final union. The application is never renewed in the foul air of a ward. *Primary* union *has* been obtained.

"After a circular amputation of the thigh, an assistant steadies the stump, while another pulls apart the edges of the divided integument, and the surgeon proceeds to fill the cavity thus presented to him, with small masses of cotton torn from the sheet of wadding, small at first and applied accurately to every part of the cut surface, then larger masses as it becomes filled, and then layers of the wadding are applied over and around the stump, and upon the hip and pelvis, and over all a spica bandage put on with great care, and as much compressing force as possible. No air must come in contact with the wound that has not filtered through the thick mass of cotton. Moreover this cotton must be of good quality, fresh from the manufactory, and it must not have been exposed to the air of the hospital."

The supporters of this new mode of dressing claim that, aside from protection against poisonous germs, it prevents inflammation, by virtue of a compression uniform and elastic, a temperature equal and favorable, and a partial immobility, and protection from external injury; that by it the growth of granulations is promoted, the pain and trouble of frequent dressing non-necessitated, and in general, a decided enhancement given the patient's progress towards recovery.

### *LODGMENT OF A FOREIGN BODY IN A STRICTURED ÆSOPHAGUS.*

Dr. Balch, of Yonkers, New York, reports in the New York *Medical Jonrnal* for March, as follows: Mr. F., æt. 27, when two and a half years of age, accidentally swallowed some sulphuric acid. The effect was most disastrous, and eventuated in two strictures, one at the upper third of the æsophagus, slight, and again at the lower third, which was the more formidable. A long struggle for life followed the swallowing of the acid, and for about six years he subsisted entirely on liquids and semi-solid food. Subsequently he essayed the use of solid diet, and as a consequence, suffered from repeated attacks of æsophageal obstruction, varying in duration from a few minutes to *fifty hours.* January 8, 1875, patient applied for relief, stating that twelve hours previously, while indulging in roast-beef, a portion thereof became lodged in his throat so firmly that not even a drop of water would pass the obstruction. Repeated trials at dislodgment by mechanical means having failed, the following presciption was ordered: ℞. Acid hydrochlor. dil., fʒij.; pepsin, ʒj.; aq. puræ q. s. ad., f℥ij. M. Teaspoonful swallowed repeatedly, the object being to digest the meat. This treatment was persevered in for thirty-seven hours from the date of the obstruction; when, after two ineffectual attempts at deglutition, a third succeeded. No untoward sequel resulted, other than a sore throat for a few days, liquids and semi-solid food being meanwhile employed. After which patient resumed his ordinary diet.

"The excessive use of mechanical means to dislodge an article of food lodged in the æsophagus, and which does not interfere with respiration, appears to me to be uncalled for, until efforts have been made to digest or dissolve it. I have been unable to find any record of cases in which a digestive, or dissolving treatment has been used. The idea suggested itself to me, and the patient said it had been employed by Dr. Parker, of Poughkeepsie, New York, in a previous attack, and with the same fortunate result."

### *EXTERNAL STIMULI IN THE TREATMENT OF OPIUM NARCOSIS.*

The history of the subjoined cases communicated to me by my friend, Dr. Thornton, of Peapack, New Jersey, are possessed of no little importance, as showing the value of certain measures, almost always available, in antagonizing, a lethal dose of the drug under consideration.

A. A., æt. five months, was given "some laudanum," to quiet restlessness; the exact amount could not be ascertained, as a studious effort at concealment was made in that direction.

Two hours subsequently, I was called to see the patient. The symptoms were so alarming, that I thought the prospect of success attending any plan of treatment were slight indeed. Extreme pallor of the whole body, lividity of the lips and tongue, muscles remarkably flaccid, and respiration very slow and irregular, not exceeding at times six per minute, and occasionally I thought breaathing had fully ceased. The circulation was very feeble, the radial pulse, part of the time, being imperceptible. Deglutition was impossible for several hours.

I confess I was a good deal puzzled, but taking what seemed a "common sense" view of the situation, I concluded the prominent indications of treatment were, to increase the activity of the circulation and determine blood to the surface, as well as to increase the frequency of the respiratory movements, and to keep them "up and doing" until the poison was considerably eliminated.

The child being unable to swallow, I was forced to rely upon a course of external treatment. Finding no benefit from the use of sinapisms, hot pediluvia, and cold to the head, I resorted to the hot mustardized bath, made by stirring half a pound of ground mustard in a pail two-thirds full of water. In this, of a temperature as high as could be borne, I immersed the child to its neck, kept it there, until the skin became thoroughly reddened. By this I succeeded most of the time in producing a well-marked increase in the activity of the respiratory movements and circulation. The immersion was repeated whenever the skin became pale, and when it failed to cause active respiratory action, I applied for a moment to the epigastrium, and over the chest, a heated metalic surface—using my metallic tobacco box,—so hot that its application was very painful, in fact, was followed by vesication. This I found to be a powerful exciter of reflex action.

This treatment was continued for three hours, when the pallor disappeared, and the respirations became regular. As soon as the patient could swallow, spts. ammon. aroma. was administered at short intervals, and the child made a perfect recovery.

The second case differed in no material respect, save the more tender age of the patient, being only four days old, and the same plan of treatment eventuated in a similar happy result.

In closing the doctor remarks, "you may think this was rough treatment. I think so too, and was really as much astonished that they recovered from it, as from the narcotism, especially in the case of the babe four days old. But, 'all is well that ends well,' and I consoled myself with the reflection that the end justified the means."

